# Integration of light and circadian signals that regulate chloroplast transcription by a nuclear‐encoded sigma factor

**DOI:** 10.1111/nph.14176

**Published:** 2016-09-15

**Authors:** Fiona E. Belbin, Zeenat B. Noordally, Sarah J. Wetherill, Kelly A. Atkins, Keara A. Franklin, Antony N. Dodd

**Affiliations:** ^1^ School of Biological SciencesUniversity of BristolBristol Life Sciences Building, 24 Tyndall AvenueBristolBS8 1TQUK; ^2^ Department of Botany and Plant BiologyUniversity of GenevaGenevaCH‐1211Switzerland; ^3^ Department of BiologyUniversity of YorkYorkYO10 5DDUK

**Keywords:** *Arabidopsis thaliana*, chloroplasts, circadian rhythms, photobiology, signal transduction

## Abstract

- We investigated the signalling pathways that regulate chloroplast transcription in response to environmental signals. One mechanism controlling plastid transcription involves nuclear‐encoded sigma subunits of plastid‐encoded plastid RNA polymerase. Transcripts encoding the sigma factor SIG5 are regulated by light and the circadian clock. However, the extent to which a chloroplast target of SIG5 is regulated by light‐induced changes in SIG5 expression is unknown. Moreover, the photoreceptor signalling pathways underlying the circadian regulation of chloroplast transcription by SIG5 are unidentified.
- We monitored the regulation of chloroplast transcription in photoreceptor and sigma factor mutants under controlled light regimes in *Arabidopsis thaliana*.
- We established that a chloroplast transcriptional response to light intensity was mediated by SIG5; a chloroplast transcriptional response to the relative proportions of red and far red light was regulated by SIG5 through phytochrome and photosynthetic signals; and the circadian regulation of chloroplast transcription by SIG5 was predominantly dependent on blue light and cryptochrome.
- Our experiments reveal the extensive integration of signals concerning the light environment by a single sigma factor to regulate chloroplast transcription. This may originate from an evolutionarily ancient mechanism that protects photosynthetic bacteria from high light stress, which subsequently became integrated with higher plant phototransduction networks.

## Introduction

Plants are sessile autotrophs that require light for photosynthesis within chloroplasts, but experience continuous changes in their light environment. Predictable changes in light conditions arise from day–night cycles, and unpredictable changes include the effects of weather and shading by competitors. Phototransduction pathways and circadian clocks allow plants to anticipate, sense and respond to these environmental changes.

Both predictable and unpredictable changes in light conditions are perceived by photoreceptors, including phytochromes, cryptochromes, phototropins, other blue light‐sensing light‐oxygen‐voltage (LOV)‐domain photoreceptors and the UV‐B photoreceptor UV RESISTANCE LOCUS8 (UVR8) (Casal, 2013). These elicit changes in gene expression that underlie global alterations in development and physiology (Casal, 2013). The action spectra of photoreceptors are allied closely with the wavelengths of light that are available for photosynthesis (Rockwell *et al*., 2014), because photoreceptors regulate physiology and development to optimize photosynthetic light harvesting. Phototransduction pathways also synchronize the plant circadian oscillator with the day–night cycles of the environment (Somers *et al*., 1998). The plant circadian oscillator comprises a network of interlocked transcription/translation feedback loops that produce a cellular estimate of the time of day (Nagel & Kay, 2012), which increases growth and fitness (Harmer *et al*., 2000; Dodd *et al*., 2005; Michael *et al*., 2008).

Chloroplast transcription is regulated by light and the circadian clock (Gamble & Mullet, 1989; Klein & Mullet, 1990; Tsinoremas *et al*., 1996; Noordally *et al*., 2013), but knowledge of the mechanisms that integrate these signals is incomplete. Chloroplast genes are transcribed by two types of RNA polymerase: plastid‐encoded plastid RNA polymerase (PEP) and nuclear‐encoded plastid RNA polymerase (NEP) (Kanamaru *et al*., 1999). PEP requires a bacterial‐type σ^70^ subunit (sigma factor) to confer promoter specificity and initiate transcription. In higher plants, six sigma factors are encoded by the nuclear genome. It is thought that, during higher plant evolution, sigma factors transferred from the genomes of ancestral chloroplasts to the nuclear genome, and provide a mechanism for nuclear control of the specificity of chloroplast transcription (Kanamaru *et al*., 1999; Ueda *et al*., 2013).

Transcripts encoding *SIGMA FACTOR5* (*SIG5*) are regulated by several light signals in mature leaves (Ichikawa *et al*., 2008; Onda *et al*., 2008; Mellenthin *et al*., 2014) and during de‐etiolation (Monte *et al*., 2004; Tepperman *et al*., 2006). This involves the cryptochrome, phytochrome and UVR8 photoreceptors (Monte *et al*., 2004; Brown & Jenkins, 2008; Onda *et al*., 2008; Mellenthin *et al*., 2014). *SIG5* transcript abundance is also regulated by photosynthesis (Mellenthin *et al*., 2014), abiotic stress (Nagashima *et al*., 2004), retrograde signalling (Ankele *et al*., 2007) and the circadian clock (Noordally *et al*., 2013). Within chloroplasts, SIG5 regulates transcription of the blue light‐responsive promoter (BLRP) of *psbD* (*psbD* BLRP), which encodes the light‐labile D2 protein of photosystem II (PSII) (Nagashima *et al*., 2004), and transcripts with less well‐characterized promoters (Noordally *et al*., 2013). *psbD* BLRP is one of at least four differently sized transcripts that originate from the chloroplast *psbDC* operon in Arabidopsis (Hoffer & Christopher, 1997; Hanaoka *et al*., 2003; Nagashima *et al*., 2004). Here, we focused on *psbD* BLRP because it provides an experimentally tractable readout of chloroplast transcriptional regulation by SIG5.

Although sigma factors are known to be regulated by a variety of light signals, the extent to which this alters the transcription of sigma factor‐regulated genes within chloroplasts is not known. We investigated this using nuclear‐encoded SIG5 and chloroplast‐encoded *psbD* BLRP as a model. First, we report a series of new findings concerning the regulation of chloroplast transcription and the sigma factor SIG5 by light. Second, we demonstrate that specific light signalling pathways are required for SIG5 to maintain circadian rhythms of transcription of chloroplast *psbD* BLRP. We conclude that sigma factors integrate and communicate several types of information concerning the light environment to the chloroplast genome.

## Materials and Methods

### Plant materials and growth conditions

Seeds of *Arabidopsis thaliana* (L.) Heynh. were surface sterilized by exposure to 70% (v/v) ethanol for 1 min, 20% (v/v) domestic bleach for 12 min and then washed twice with sterile distilled H_2_O. Seeds were resuspended in 0.1% (w/v) agar and sown individually onto half‐strength (2.15 g l^−1^) Murashige and Skoog nutrient mix (basal salts without vitamins, pH 6.8; Duchefa Biochimie, Haarlem, the Netherlands) in 0.8% (w/v) agar, without sucrose supplementation. For luciferase imaging, seeds were sown into sterile plastic rings embedded within growth medium (15 seeds per ring) to produce circular regions of luciferase bioluminescence (Love *et al*., 2004; Noordally *et al*., 2013; Dodd *et al*., 2014). Seeds were stratified in the dark for 3 d at 4°C and then cultivated under 12 h : 12 h, light : dark cycles at 19°C and 90 μmol m^−2^ s^−1^ white light (MLR‐352; Panasonic, Osaka, Japan). Modified conditions were required for comparable germination and growth of *phyABCDE* mutants, involving germination in 120 μmol m^−2^ s^−1^ white light (Microclima 1600E; Snijder Scientific, Tillburg, the Netherlands) at 20°C with 16 h : 8 h, light : dark cycles for 5 d, before transfer to standard growth conditions (as earlier). All photoreceptor mutants described, except *phyABCDE*, were transformed with *SIG5::LUCIFERASE*+ (Noordally *et al*., 2013). T_3_ generation *SIG5::LUCIFERASE*+‐expressing homozygous seedlings were used for all experimentation. Multiple transgenic lines were screened to identify those having comparable luciferase bioluminescence, and then characterized using bioluminescence time course imaging to select lines for experimentation with representative circadian periods (Supporting Information Fig. S1). Eleven‐day‐old seedlings were used for all experiments.

Genotypes were Col‐0, Landsberg *erecta* (L. *er*), *sig5‐*3 (Noordally *et al*., 2013), *phyA‐201* (Nagatani *et al*., 1993), *phyB‐*5 (Nagatani *et al*., 1993), *phyA*‐201 *phyB*‐5 (Reed *et al*., 1994), *phyABCDE* (Hu *et al*., 2013), *cry1*‐B104 (Bruggemann *et al*., 1998), *cry2*‐1 (Guo *et al*., 1998), *cry1 cry2* (*hy4*‐1 *fha*‐1, El‐Assal *et al*., 2003).

### Transcript abundance

Aerial tissue was harvested 11 d after germination, as described previously (Noordally *et al*., 2013). Total RNA was extracted using a NucleoSpin RNA extraction kit (Macherey‐Nagel, Duren, Germany), from which cDNA was synthesized (High Capacity cDNA Reverse Transcription Kit, ThermoFisher, Waltham, MA, USA). Quantitative reverse transcription‐polymerase chain reaction (qRT‐PCR) analysis was performed using Brilliant III Ultra‐Fast SYBR Green qRT‐PCR Master Mix (Agilent Technologies, Santa Clara, CA, USA, using Agilent Mx3005P qRT‐PCR instruments) and the primers described later. Transcript abundance was relative to *ACTIN2* (*ACT2*), an established reference for the study of this pathway (Noordally *et al*., 2013), and calculated using the ΔΔ*C*t method. For light induction experiments, transcript abundance was measured 1 h (*SIG5*) and 4 h (*psbD* BLRP) after the start of light treatments, as a time delay exists between the upregulation of *SIG5* and *psbD* BLRP transcripts (Noordally *et al*., 2013), and these times correspond with maximum *SIG5* and *psbD* BLRP transcript abundance attained after exposure to light of dark‐adapted seedlings (Mochizuki *et al*., 2004; Onda *et al*., 2008; Noordally *et al*., 2013). qRT‐PCR primers were *SIG5* (GTGTTGGAGCTAATAACAGCAGACA (FP), TGTCGAATAACCAGACTCTCTTTCG (RP)); *psbD* BLRP (GGAAATCCGTCGATATCTCT (FP), CTCTCTTTCTCTAGGCAGGAAC (RP)) (Mochizuki *et al*., 2004); *LHY* (*LATE ELONGATED HYPOCOTYL*) (ACGAAACAGGTAAGTGGCGACA (FP), TGGGAACATCTTGAACCGCGTT (RP)) (Noordally *et al*., 2013); *ACT2* (TCAGATGCCCAGAAGTGTTGTTCC (FP), CCGTACAGATCCTTCCTGATATCC (RP), or TGAGAGATTCAGATGCCCAGAA (FP), TGGATTCCAGCAGCTTCCAT (RP) in Fig. 4(c) only (see later)).

### Light conditions

Blue (B), red (R) and far red (FR) light manipulations used custom LED panels installed within temperature‐controlled growth chambers, and custom Photek LB‐1 R/FR/B LED panels controlled by the bioluminescence imaging system. Photosynthetically active radiation (PAR) and light spectra were quantified with a spectroradiometer (Ocean Optics, Dunedin, FL, USA). Peak output wavelengths of R, B and FR LEDs were 660, 470 and 740 nm, respectively (Fig. S2). The R : FR ratio was calculated using PAR integrated from 660 to 670 nm divided by 725–735 nm (Franklin, 2008). Light induction experiments used 25 μmol m^−2^ s^−1^ total photon flux density (PFD) for each light colour treatment, except Fig. 4(c) only (see later), which used 10 μmol m^−2^ s^−1^ per treatment. In all figures, ***, *P* < 0.001; **, *P* < 0.01; *, *P* < 0.05; ns, not statistically significant. All light treatments commenced at zeitgeber time (ZT) 4, using dark‐adapted seedlings, because *SIG5* has greatest sensitivity to B light pulses at ZT4 (Noordally *et al*., 2013).

### Bioluminescence imaging

Clusters of 10‐d‐old seedlings surrounded by sterile rings (e.g. Fig. S3) were dosed with 100 μl of 5 mM luciferin (potassium salt of D‐luciferin; Melford Laboratories Ltd, Ipswich, UK) 24 h before imaging. Bioluminescence was measured using a Lumintek EM‐CCD imaging system (Photek Ltd, St Leonards on Sea, UK) controlled by Image32 software (Photek) and custom control scripts (45‐s integrations, EM gain setting 2700). For experiments investigating *SIG5::LUCIFERASE* induction by light, 11‐d‐old seedlings were exposed to the light regime specified after dark adaptation for 24 h. Images were captured at 13‐min intervals, preceded by a dark delay of 2 min to eliminate chlorophyll autofluorescence from the bioluminescence signal. Sequences of images lasted between 4 and 8 h, depending on the experiment; data on the figures represent peak *SIG5::LUCIFERASE* activity. Circadian time course imaging of *SIG5::LUCIFERASE* bioluminescence commenced at ZT0, using 11‐d‐old seedlings entrained previously to 12 h : 12 h, light : dark cycles. Seedlings were exposed to two 12 h : 12 h, light : dark cycles of the wavelength(s) under investigation before transfer to constant light, to reduce transitory effects. Bioluminescence images were captured approximately every hour. Imaging data were analysed using Image32 software (Photek), with circadian time courses analysed further using the fast Fourier transform‐nonlinear least‐squares (FFT‐NLLS) algorithm within Brass software (Southern & Millar, 2005), downloaded in 2015 from http://millar.bio.ed.ac.uk. The first 24 h of data in constant light were discarded before FFT‐NLLS analysis to remove transient responses to the final dark period.

### Inhibitor experiments

For experiments with norflurazon (Sigma‐Aldrich), growth medium was supplemented with 5 μM norflurazon and 1% (w/v) sucrose to allow growth in the absence of photosynthesis (e.g. Fig. S3a). For bioluminescence imaging experiments with 3‐(3,4‐dichlorophenyl)‐1,1‐dimethylurea (DCMU, Sigma‐Aldrich), 20 μM DCMU was added to the 100 μl of 5 mM luciferin that was dosed onto seedlings. For RNA sampling, 100 μl of 20 μM DCMU was dosed onto seedlings. In both cases, DCMU was dosed onto seedlings 24 h before the start of light treatment. Inhibitors were dissolved in dimethylsulfoxide (DMSO) (working concentrations of DMSO were 0.0025% (v/v) and 0.01% (v/v) with norflurazon and DCMU, respectively), and inhibitor controls contained an equal volume of DMSO without the inhibitor.

## Results

**Figure 1:**
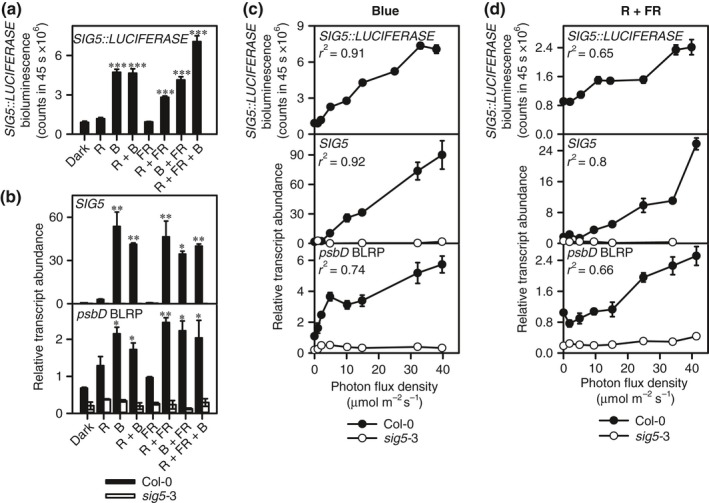
Signalling of information concerning light intensity to the chloroplast genome by nuclear‐encoded SIGMA FACTOR5 (SIG5) in *Arabidopsis thaliana*. (a, b) Relative induction of (a) *SIG5::LUCIFERASE* and (b) *SIG5* and *psbD* blue light‐responsive promoter (BLRP) transcripts under several light conditions in dark‐adapted wild‐type and *sig5*‐3 seedlings (light conditions: R, red light; B, blue light; FR, far red light; and combinations of these with 25 μmol m^−2^ s^−1^ of each wavelength). (c, d) The magnitude of induction of *SIG5::LUCIFERASE* and the abundance of *SIG5* and *psbD* BLRP transcripts were dependent on light intensity under (c) blue light and (d) red and far red light (R : FR = 0.7). All light treatments commenced at zeitgeber time 4. (a, b) ANOVA and *post‐hoc* Tukey analysis compared each light condition with dark control. ***, *P* < 0.001; **, *P* < 0.01; *, *P* < 0.05. (c, d) *r* ^2^ from linear regression. *y* axes in (c, d) are not comparable because experiments were analysed separately. Data are mean ± SE:* n* = 2–6 (transcript abundance); *n* = 5–12 (*SIG5::LUCIFERASE*).

We used the regulation of chloroplast *psbD* BLRP by nuclear‐encoded SIG5 as an experimental model. To provide a basis for subsequent experiments, we investigated the accumulation of chloroplast *psbD* BLRP transcripts in wild‐type and *sig5*‐3 loss‐of‐function plants, under various light conditions, to determine the role of nuclear‐encoded SIG5 in the regulation of chloroplast *psbD* BLRP by light. Like *SIG5* transcripts, *SIG5* promoter activity and chloroplast‐encoded *psbD* BLRP transcripts were induced most strongly by B light, other treatments including B light, and a combination of R and FR light with R : FR = 0.7 (Fig. 1a,b). *SIG5* transcripts and *SIG5* promoter activity were not induced by either R or FR light alone (Fig. 1a,b). The transcriptional responses of *psbD* BLRP were SIG5 dependent because light treatments did not induce *psbD* BLRP in the s*ig5*‐3 loss‐of‐function mutant (Fig. 1b). The behaviour of *SIG5* transcripts (Fig. 1b) was consistent with studies conducted under similar conditions (Mochizuki *et al*., 2004; Nagashima *et al*., 2004; Onda *et al*., 2008; Noordally *et al*., 2013). The regulation of *SIG5* promoter activity by light, measured with *SIG5::LUCIFERASE*, appeared to account largely for the regulation of *SIG5* transcript accumulation (Fig. 1a,b).

### SIG5 communicates information concerning light intensity and quality to chloroplasts

We hypothesized that chloroplast transcription is regulated by SIG5 in response to light intensity, as: (1) *SIG5* transcript abundance depends on B light intensity (Onda *et al*., 2008); (2) *psbD* BLRP is regulated by SIG5 in a dose‐dependent manner (Onda *et al*., 2008); and (3) we found that both B and R + FR light upregulation of *psbD* BLRP was dependent on SIG5 (Fig. 1b). It is not known whether *SIG5* transcription is dependent on the intensity of R light, nor how these fluence responses of *SIG5* affect chloroplast transcription. To test this, we applied a range of intensities of either B or R + FR light to seedlings. Treatment with each light intensity commenced at ZT4, using separate batches of seedlings (we did not progressively increase light intensity over time, because that approach would be confounded by circadian gating). In both B and R + FR light, the magnitude of induction of chloroplast *psbD* BLRP transcripts was determined by PFD, and also required SIG5 (Fig. 1c,d). The magnitude of induction of the *SIG5* promoter and *SIG5* transcript abundance were also determined by PFD (Fig. 1c,d). This suggests that, across the PFD range investigated, regulation of the *SIG5* promoter by PFD of both B and R + FR light controlled the accumulation of *SIG5* transcripts, causing the magnitude of chloroplast *psbD* BLRP transcript accumulation to be PFD dependent.

### Photoreceptors and retrograde signals underlie the regulation of chloroplast transcription in response to light intensity by SIG5

Plant responses to light, including the transcription of SIG5, are mediated by photoreceptors and photosynthesis (Onda *et al*., 2008; Mellenthin *et al*., 2014). It is not known which of these light response pathways underlies the light intensity‐dependent transcriptional response that we identified for *SIG5* and chloroplast *psbD* BLRP (Fig. 1c,d). Therefore, we investigated this question with a combination of photoreceptor mutants and photosynthetic inhibitors. Regulation of *SIG5* and *psbD* BLRP has been reported to involve the photoreceptors phytochromeA (phyA), cryptochrome1 (cry1) and cry2 (Thum *et al*., 2001; Ichikawa *et al*., 2004; Onda *et al*., 2008; Mellenthin *et al*., 2014). Although phyA was required for *SIG5* induction by R + FR light and phyB may suppress *SIG5* transcript accumulation (Fig. S4b), *SIG5* was not regulated by R or FR light when applied alone (Fig. 1a,b; Mochizuki *et al*., 2004; Onda *et al*., 2008; Noordally *et al*., 2013). A single report demonstrating SIG5 induction in de‐etiolated seedlings by R or FR light alone used sucrose‐supplemented growth media (Mellenthin *et al*., 2014). *SIG5* transcripts were induced by B light in the *phyA* mutant, presumably as a result of cryptochrome‐mediated regulation of *SIG5* (Fig. S4d). However, as the regulation of SIG5 by R and FR light is atypical for phytochrome signalling, we reasoned that additional mechanisms act alongside phytochromes to regulate chloroplast transcription by SIG5 in response to the intensity of R + FR light.

**Figure 2:**
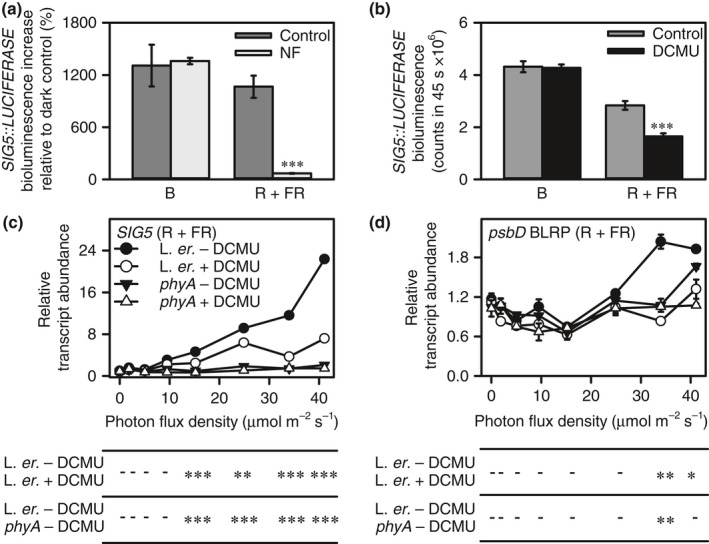
Photosynthetic and phytochrome signals in *Arabidopsis thaliana* underlie SIGMA FACTOR5 (SIG5)‐mediated signals to chloroplasts concerning red (R)/far red (FR) light intensity. (a, b) *SIG5::LUCIFERASE* induction by blue (B) and R + FR light in the presence of norflurazon (NF) or 3‐(3,4‐dichlorophenyl)‐1,1‐dimethylurea (DCMU), using 25 μmol m^−2^ s^−1^ of each wavelength. (a) Represented as proportional increase in bioluminescence relative to dark control (because inclusion of sucrose within growth media to allow growth in the presence of norflurazon, and cultivation on norflurazon, changed baseline bioluminescence). (c, d) Light intensity response of *SIG5* and *psbD* blue light‐responsive promoter (BLRP) transcripts in R + FR light in wild‐type (WT) and *phyA*‐201 with DCMU supplementation. All light treatments commenced at zeitgeber time 4 (ZT4). Significance determined by (a, b) *t*‐tests and (c, d) ANOVA and Tukey *post‐hoc* analysis, comparing WT with and without DCMU and WT with DCMU and *phyA*‐201 without DCMU. (a–d) ***, *P* < 0.001; **, *P* < 0.01; *, *P* < 0.05. (c, d) Hyphen indicates not statistically significant. Data are mean ± SE;* n* = 2–6 (quantitative reverse transcription‐polymerase chain reaction, qRT‐PCR); *n* = 5–12 (*SIG5::LUCIFERASE*). R + FR, combination of red and far red light (R : FR = 0.7).

We investigated the involvement of retrograde signalling in the control of chloroplast transcription by SIG5 in response to PFD. B light activation of *SIG5::LUCIFERASE* was unaltered by norflurazon, which inhibits carotenoid biosynthesis, leading to photobleaching (e.g. Fig. S3a). By contrast, norflurazon inhibited the upregulation of *SIG5::LUCIFERASE* by R + FR light (Figs 2a, S3a). We also investigated the effect of DCMU, an inhibitor of photosynthetic electron transport between PSII and plastoquinone (PQ), on light activation of SIG5‐mediated signals to chloroplasts. First, we determined the minimum effective dose for the inhibition of photosynthesis by DCMU under our experimental conditions using modulated PSII chlorophyll fluorescence (Imaging‐PAM M, Walz, Germany). Seedlings grown exactly as for bioluminescence imaging and RNA sampling were dosed with 0, 5, 10, 15, 20, 35 or 50 μM DCMU (mixed with and without luciferin for Col‐0 *SIG5::LUCIFERASE* and L. *er*., respectively) and dark adapted for 24 h before determination of *F*_o_ and *F*_m_ (intensity setting 1, frequency 4). Actinic light (107 μmol m^−2^ s^−1^) was switched on for 10 min, after which the effective quantum yield of PSII (Y(II)) was calculated as (*F*_m_′ – *F*′)/*F*_m_′, where *F*_m_′ is the maximum fluorescence emission from the light‐adapted seedling after a saturating pulse, and *F*′ is the chlorophyll fluorescence emission from light‐adapted seedlings. Based on these data, we used DCMU at a concentration of 20 μM, and luciferin did not alter the efficacy of DCMU.

DCMU treatment reduced R + FR light induction of *SIG5::LUCIFERASE* by 42%, whereas induction of *SIG5::LUCIFERASE* by B light was insensitive to DCMU (Fig. 2b). Together, these results indicated that a retrograde signal arising from photosynthetic electron transport was required for the regulation of the *SIG5* promoter by R + FR but not B light.

We used this information to investigate the contribution of phytochrome and photosynthetic signals to the regulation of chloroplast transcription in response to the intensity of R + FR light. There was some variation in the sensitivity of *psbD* BLRP transcripts to R + FR light; the PFD threshold for significant *psbD* BLRP upregulation by R + FR light was 25 μmol m^−2^ s^−1^ in Fig. 1(d) (*P* = 0.001) and 35 μmol m^−2^ s^−1^ in Fig. 2(d) (*P* = 0.008; two‐sample *t*‐tests relative to dark controls). *SIG5* and *psbD* BLRP were generally not induced significantly in *phyA* mutants at any PFD relative to dark controls (Fig. 2c,d), demonstrating that this response of *SIG5* to light intensity was dependent on phyA. A single exception was that, in *phyA*,* psbD* BLRP was induced by R + FR light at 40 μmol m^−2^ s^−1^ in the absence of DCMU, and this response was abolished when DCMU was added (Fig. 2d). Across the PFD range tested, DCMU reduced the slope estimate (*r* ^2^) of the R + FR PFD response of *SIG5* from 0.46 to 0.14, and of *psbD* BLRP from 0.03 to 0.01 (Fig. 2c,d). The absence of an effect of DCMU on B light activation of *SIG5::LUCIFERASE* (Fig. 2b) suggests that the DCMU sensitivity of R + FR light induction of *SIG5::LUCIFERASE* is a specific signalling response rather than a nonspecific consequence of DCMU‐induced oxidative damage. Overall, these data indicate that, although R + FR light activation of *psbD* BLRP by SIG5 is dependent on phyA, a photosynthetic signal underlies the quantitative response of the pathway to R + FR light intensity.

### Regulation of SIG5‐mediated signalling to chloroplasts by the proportions of red and far red light

As R or FR light alone had little effect on chloroplast *psbD* BLRP transcription by SIG5, but R and FR light in combination induced this pathway (Fig. 1a,b), we reasoned that chloroplast *psbD* BLRP might be regulated by the relative proportions of R and FR light in a SIG5‐dependent manner. In nature, R : FR light conditions provide plants with information concerning vegetational shade or the threat of vegetational shade, because vegetation absorbs R light and transmits and reflects FR light. The balance of R and FR light also affects plants because R and FR light preferentially excite PSII and PSI, respectively, altering the energy balance across the photosynthetic electron transport system and the redox state of the PQ pool (Pfannschmidt *et al*., 1999; Bonardi *et al*., 2005).

**Figure 3:**
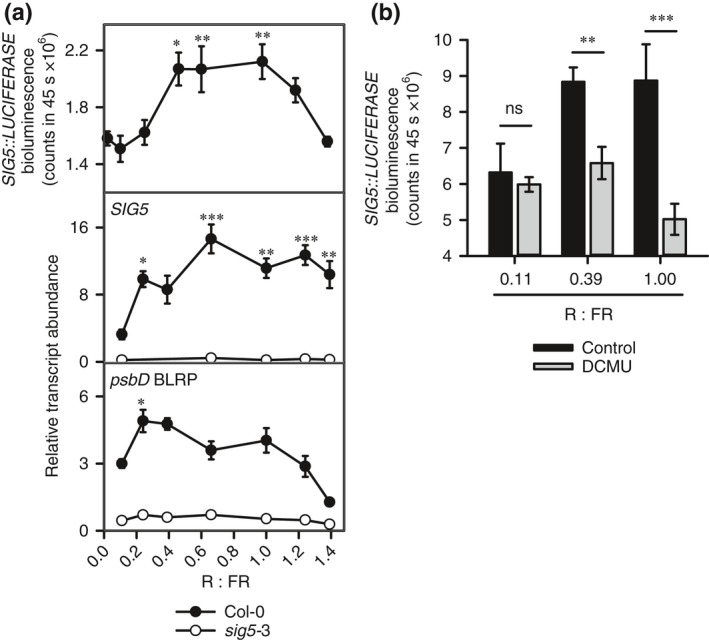
Relative proportions of red (R) and far red (FR) light regulate SIGMA FACTOR5 (SIG5)‐mediated signalling to chloroplasts in *Arabidopsis thaliana*. (a) *SIG5::LUCIFERASE* bioluminescence and abundance of transcripts encoding *SIG5* and *psbD* blue light‐responsive promoter (BLRP) in dark‐adapted seedlings exposed to a range of R : FR ratios (totalling 25 μmol m^−2^ s^−1^). (b) Role of photosynthesis in R : FR response of *SIG5::LUCIFERASE* in seedlings treated with 3‐(3,4‐dichlorophenyl)‐1,1‐dimethylurea (DCMU). Analysis by (a) ANOVA and *post‐hoc* Tukey test comparing lowest R : FR with all other R : FR; (b) two‐sample *t*‐tests within each R : FR, where: ***, *P* < 0.001; **, *P* < 0.01; *, *P* < 0.05; ns, not significant. Data are mean ± SE;* n* = 5–12 (*SIG5::LUCIFERASE*); *n* = 2–6 (quantitative reverse transcription‐polymerase chain reaction, qRT‐PCR). Light treatments commenced at zeitgeber time 4.

It is not known whether sigma factor‐mediated signals to chloroplasts are regulated by the relative proportions of R and FR light. To test this, we exposed dark‐adapted seedlings to R : FR light conditions in the range 0.02–1.4, and monitored both *SIG5* promoter activity and *SIG5* and *psbD* BLRP transcript abundance (Fig. 3a). The magnitude of activation of *SIG5*, its promoter and chloroplast‐encoded *psbD* BLRP was dependent on the relative proportions of R and FR light (Fig. 3a). *SIG5::LUCIFERASE* was induced strongly by R : FR in the range 0.46–0.96, and *SIG5* transcripts were induced most strongly by R : FR in the range 0.66–1.24 (Fig. 3a). *psbD* BLRP induction was reduced at very low R : FR and R : FR exceeding 1.2 (Fig. 3a). The *psbD* BLRP response to R : FR conditions was dependent on SIG5, as *psbD* BLRP was not induced in *sig5*‐3 (Fig. 3a). This was consistent with Fig. 1(a,b), where *SIG5* and *psbD* BLRP transcript accumulation was low under R or FR light alone, but high under R + FR light (R + FR = R : FR conditions of 0.7).

phyA promoted *SIG5* transcription under R + FR light (Fig. S4c), but the smallest induction of *SIG5::LUCIFERASE* and *SIG5* transcript abundance occurred under conditions of very low R : FR (Fig. 3a), when phyA signalling would be expected to be greatest (Martínez‐García *et al*., 2014). We explored this difference by testing the contribution of photosynthesis to R : FR responses of *SIG5*. DCMU had no effect on the small increase in *SIG5::LUCIFERASE* at low R : FR, yet inhibited *SIG5::LUCIFERASE* upregulation at higher R : FR conditions (Fig. 3b). The magnitude of *SIG5::LUCIFERASE* induction was dependent on the proportions of R and FR light, rather than simply R light intensity, because *SIG5* was not induced by R light alone (Fig. 1a,b).

### Circadian signalling to chloroplasts by SIG5 is primarily dependent on blue light and cryptochrome

SIG5 communicates circadian timing information from the nuclear‐encoded circadian oscillator to circadian‐regulated chloroplast transcripts, including *psbD* BLRP (Nakahira *et al*., 1998; Ichikawa *et al*., 2008; Noordally *et al*., 2013). Specific light conditions and photoreceptors regulate SIG5 induction of *psbD* BLRP in dark‐adapted seedlings (Figs 1, S4), the circadian clock gates transient B light induction of *SIG5* and *psbD* BLRP (Noordally *et al*., 2013) and, in cycles of B light and darkness, cryptochromes contribute to the transcriptional patterns of a *SIG5* orthologue in *Physcomitrella* (Ichikawa *et al*., 2004). It is not known which photoreceptor systems or light conditions underlie SIG5‐mediated circadian signalling to chloroplasts, and so we investigated this with a combination of photoreceptor mutants and manipulations to the light conditions.

**Figure 4:**
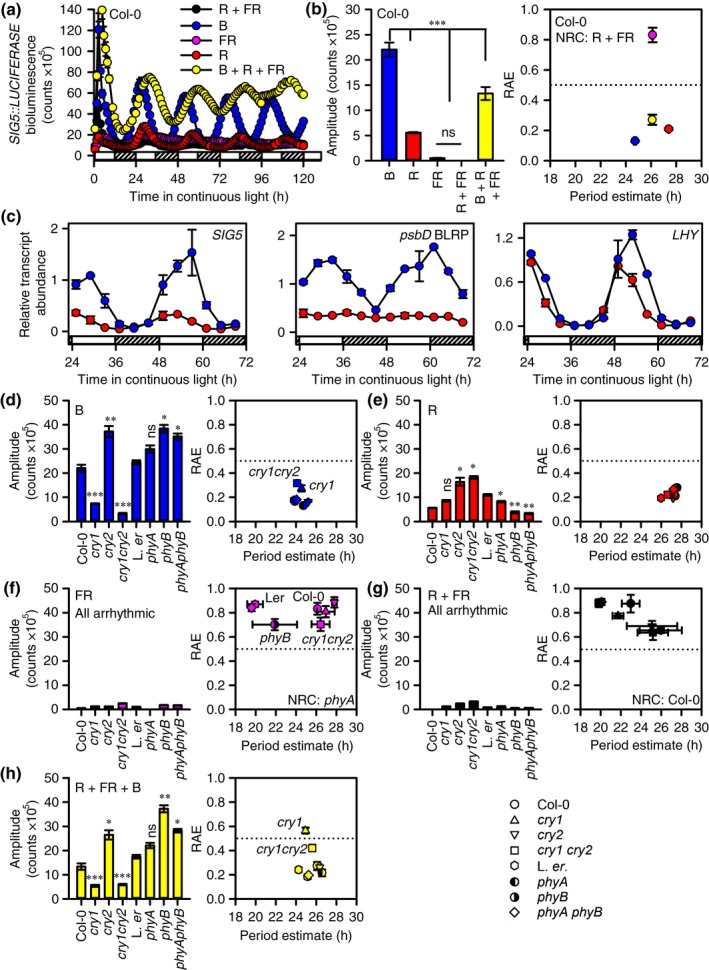
Blue (B) light and cryptochrome photoreceptors play major roles in SIGMA FACTOR5 (SIG5)‐mediated circadian signalling to chloroplasts in *Arabidopsis thaliana*. Circadian oscillations of (a, b) *SIG5::LUCIFERASE* under five light regimes and (c) *SIG5*,* psbD* blue light‐responsive promoter (BLRP) and *LHY* transcripts in continuous B and red (R) light. (d–h) Properties of circadian oscillations of *SIG5::LUCIFERASE* in photoreceptor mutants under five light regimes (Col‐0 and L. *er*. backgrounds for *cry* and *phy*, respectively). Hatched bars in (a, c) indicate subjective darkness. Data are mean ± SE;* n* = 4 circular clusters of seedlings; significance determined by ANOVA and Tukey *post‐hoc* analysis. Statistical significance is indicated for comparisons of (b) all treatments and (d–h) each mutant against its background, except (f) and (h) where all genotypes had low rhythmic robustness, making amplitude comparisons uninformative. ***, *P* < 0.001; **, *P* < 0.01; *, *P* < 0.05; ns, not statistically significant. Light conditions: R, red light; B, blue light; FR, far red light (and combinations of these with 25 μmol m^−2^ s^−1^ of each wavelength). NRC, no rhythmic components detected; RAE, relative amplitude error derived from analysis by fast Fourier transform‐nonlinear least‐squares method.

Circadian oscillations of *SIG5*::*LUCIFERASE* showed greatest amplitude under continuous B light, lower amplitude under a combination of B, R and FR light, and very low amplitude under continuous R light (Figs 4a,b, S5; see also Fig. S6 for these light conditions plotted separately for Col‐0). The relative amplitude error (RAE) from analysis by FFT‐NLLS indicates the quality of fit of a sine wave to the experimental data, from 0 (perfect fit) to 1 (no fit), where > 0.5 typically reflects arrhythmicity (Xu *et al*., 2007) (Fig. 4b). Using this measure, *SIG5::LUCIFERASE* was arrhythmic under both R + FR and FR light alone (Figs 4a,b, S6), but was rhythmic when B light was added to R + FR (Fig. 4a). *SIG5::LUCIFERASE* has been shown elsewhere to be rhythmic under R + B light (Noordally *et al*., 2013). Together, these data indicate that robust circadian oscillations of the *SIG5* promoter require B light.

To determine the relationship between the arrhythmia of *SIG5::LUCIFERASE* under R and FR light and circadian oscillator function, we monitored circadian oscillations of *CCA1::LUCIFERASE* under combinations of R and FR light (Fig. S5b). *CCA1::LUCIFERASE* was rhythmic under R and R + FR light, but arrhythmic under FR light alone (Fig. S5b). The amplitude of oscillations of *CCA1::LUCIFERASE* was approximately six‐fold greater under R than R + FR light (Fig. S5b).

Next, we investigated the role of selected wavelengths in the circadian regulation of *SIG5* and *psbD* BLRP transcripts (Fig. 4c). *SIG5* and *psbD* BLRP were rhythmic under B light and, under R light, there were low‐amplitude oscillations of *SIG5* but *psbD* BLRP was arrhythmic (Fig. 4c). Circadian oscillations of *LHY* indicated that the circadian oscillator remained rhythmic under R light (Fig. 4c). It has been proposed that there is a minimum abundance of *SIG5* transcripts that is required for circadian oscillations of *psbD* BLRP (Noordally *et al*., 2013), and so the low‐amplitude oscillations of *SIG5* transcript abundance under R light may have been below this threshold for *psbD* BLRP transactivation (Fig. 4c).

We investigated the photoreceptors that underlie circadian oscillations of *SIG5::LUCIFERASE*. Under continuous B light, the amplitude of circadian oscillations of *SIG5::LUCIFERASE* was reduced substantially in *cry1* and *cry1 cry2* relative to the wild‐type (Figs 4d, S5), indicating that circadian oscillations of *SIG5* in B light were predominantly dependent on cry1. Under R light, the circadian amplitude of *SIG5::LUCIFERASE* was reduced slightly, but significantly, relative to the wild‐type in *phyA*,* phyB* and *phyA phyB* (Fig. 4e). This suggests that phyA and phyB made small contributions to the amplitude of circadian oscillations of *SIG5* promoter activity, but were not essential for its rhythmicity. *SIG5::LUCIFERASE* was arrhythmic in all genotypes in FR and R + FR light (Fig. 4f,g). Under R + FR + B light, circadian oscillations of *SIG5::LUCIFERASE* required cry1, because *SIG5::LUCIFERASE* was arrhythmic in *cry1* and had reduced rhythmic robustness in *cry1 cry2* (RAE = 0.42 ± 0.3; Fig. 4h) relative to other treatments. The greater amplitude of *SIG5::LUCIFERASE* oscillations in *cry2* relative to the wild‐type in B and R + FR + B light suggests that there was antagonism between cry1 and cry2 in the circadian regulation of *SIG5* (Fig. 4d,h). In the presence of B light, phyA and phyB appeared to antagonize the circadian amplitude of *SIG5::LUCIFERASE* oscillations (Fig. 4d,h), possibly explaining why *SIG5::LUCIFERASE* had lower circadian amplitude in R + FR + B than B light alone (Fig. 4d,h).

**Figure 5:**
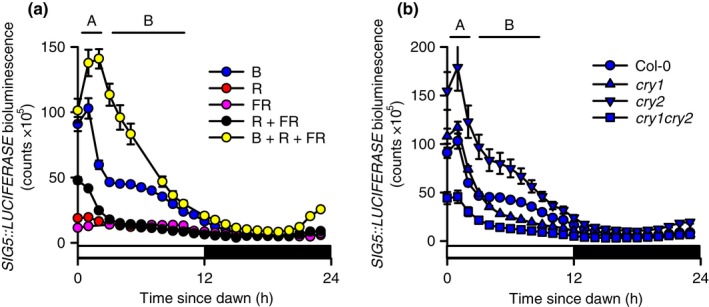
Comparison of *SIG5::LUCIFERASE* dynamics under light–dark cycles of five light conditions in (a) the Col‐0 background and (b) cryptochrome mutants of *Arabidopsis thaliana*. White and black bars on abscissa indicate light and dark periods, respectively. ‘A’ and ‘B’ above the graphs indicate two components within the *SIG5::LUCIFERASE* dynamics under monochromatic blue light. Data are mean ± SE;* n* = 4. Light conditions: R, red light; B, blue light; FR, far red light (and combinations of these with 25 μmol m^−2^ s^−1^ of each wavelength).

The dynamics of *SIG5::LUCIFERASE* under light–dark cycles of five light conditions revealed two features within the daily regulation of the *SIG5* promoter under B light (Fig. 5). Under light–dark cycles of B light, SIG5 promoter activity was induced rapidly following dawn (Fig. 5a, feature marked ‘A’), with a second more slowly acting feature present during the middle of the photoperiod (Fig. 5a, feature marked ‘B’). The ‘spike‐shoulder’ dynamics were absent from the daily regulation of *SIG5* transcription under other light conditions tested (Fig. 5a). Under B light–dark cycles, the more slowly acting feature was absent in the *cry1* and *cry1 cry2* mutants, but present in *cry2* (Fig. 5b), suggesting that the feature arose from cry1 activity. In addition, under light–dark cycles, there was clear anticipation of dawn by *SIG5::LUCIFERASE* under B + R + FR light conditions, but this was absent under B light alone (Fig. 5).

## Discussion

We present new information concerning a mechanism that integrates light and circadian cues to regulate chloroplast transcription. We first examined the dynamics of this pathway during transition from dark to light, and subsequently investigated the involvement of light conditions in circadian regulation of the pathway. Previous studies have demonstrated that nuclear‐encoded transcripts of the chloroplast RNA polymerase subunit SIG5 are induced in Arabidopsis by B light, R light and UV‐B (Monte *et al*., 2004; Brown & Jenkins, 2008; Onda *et al*., 2008; Mellenthin *et al*., 2014). Likewise, orthologues of Arabidopsis *SIG5* in rice, *Physcomitrella* and *Marchantia* are light induced (Ichikawa *et al*., 2004; Kubota *et al*., 2007; Kanazawa *et al*., 2013). Although the sigma factor SIG5 appears to be conserved amongst land plants (Kanazawa *et al*., 2013), in cyanobacteria and a species of red alga other sigma factors are light induced (Imamura *et al*., 2003; Fujii *et al*., 2015). Here, we demonstrated in Arabidopsis that light‐induced changes in sigma factor transcript abundance lead to transcriptional changes in chloroplasts in response to various light signals. We also identified specific light signalling pathways underlying the circadian regulation of chloroplast transcription by SIG5. A general interpretation is that information concerning the light environment is integrated by, and communicated to, chloroplasts by nuclear‐encoded sigma factors. SIG5 appears to communicate information to the chloroplast genome concerning light intensity and light quality (Figs 1, 3), and this information is combined with B light and cryptochrome‐dependent circadian timing cues (Fig. 4). An area for future investigation is to determine the role of the multiple transcription start sites (TSSs) within the *psbDC* operon in signal integration, as the transcription or activity of other sigma factors is regulated by light conditions and the circadian oscillator (Onda *et al*., 2008; Puthiyaveetil *et al*., 2008, 2011; Shimizu *et al*., 2010; Noordally *et al*., 2013), and other *psbDC* TSSs are light regulated depending on the developmental stage (Hoffer & Christopher, 1997).

### Circadian signalling to chloroplasts by SIG5 requires specific light signalling pathways

The circadian oscillator is rhythmic under conditions of B and R light (Somers *et al*., 1998) (see Fig. 4c for *LHY*), and so the B light dependence of circadian oscillations of *SIG5::LUCIFERASE* (Fig. 4a,c) is a specific feature of SIG5‐mediated circadian signalling to chloroplasts, rather than a dependence of the circadian oscillator on B light. By contrast, arrhythmia of *SIG5::LUCIFERASE* under continuous FR light appears to arise from arrhythmia of the circadian oscillator, as *CCA1::LUCIFERASE* was arrhythmic under these conditions (Fig. S5b), rather than representing a specific feature of the circadian regulation of SIG5. A previous report has indicated that, under continuous FR light, the circadian oscillator is rhythmic with low amplitude and altered phase (Wenden *et al*., 2011), whereas, under our experimental conditions, *CCA1::LUCIFERASE* was arrhythmic under continuous FR light (Fig. 5b). This difference could be because our experiments were conducted using sucrose‐free growth medium, whereas Wenden *et al*. (2011) included 3% sucrose in the growth medium. As FR light has been proposed to act on the circadian oscillator through the evening loop component ELF4 (Wenden *et al*., 2011) and a long‐term effect of sucrose on the circadian oscillator is mediated by the evening loop component GIGANTEA (Dalchau *et al*., 2011), phytochrome and metabolite signals may interact to provide an input to the circadian oscillator via the evening loop. Circadian oscillations of *SIG5* transcript abundance were approximately coincident with the phasing to subjective day of circadian oscillations of the promoters and transcripts of *cry1*,* cry2* and *phyA‐E* (Bognár *et al*., 1999; Tóth *et al*., 2001). However, as photoreceptor protein abundance may not cycle under constant light (Bognár *et al*., 1999; Sharrock & Clack, 2002; Mockler *et al*., 2003), rhythms of *SIG5* transcript abundance seem unlikely to be a direct consequence of oscillations of photoreceptor transcript abundance.

*cry1 cry2 SIG5::LUCIFERASE* lacked the longer circadian period identified previously for the *CHLOROPHYLL A/B‐BINDING PROTEIN2* promoter in *cry1 cry2* (Devlin & Kay, 2000). This might be explained by the temperature dependence of the period of *cry1 cry2* under conditions that include B light (Gould *et al*., 2003). Gould *et al*. (2003) indicated that the experimental temperature of Devlin & Kay (2000) (22°C) would lengthen the period of *cry1 cry2* when B and R light are present, whereas the period may be indistinguishable from the wild‐type at the lower temperature (19°C) used here (Fig. 4).

### Under light–dark cycles, SIG5 integrates several light signals that regulate chloroplast transcription

The presence of two features within the dynamics of *SIG5::LUCIFERASE* under light–dark cycles (Fig. 5a) suggests that the reduced experimental complexity provided by monochromatic B light alone (as opposed to a more complex spectrum) allowed the separation of light‐ and circadian‐regulated components of *SIG5* promoter activity. The more slowly acting feature of *SIG5::LUCIFERASE* under these conditions (marked ‘B’ on Fig. 5a, b) may be caused by circadian regulation, because this feature requires cry1 (Fig. 5b) and, under continuous B light, cry1 contributes substantially to the amplitude of circadian *SIG5::LUCIFERASE* oscillations (Fig. 4d,h).

There are several possible explanations for the lack of dawn anticipation by *SIG5::LUCIFERASE* under B light–dark cycles, compared with clear anticipation of dawn under R + FR + B light (Fig. 5). The degree of dawn anticipation by circadian reporters under light–dark cycles can reflect differences in circadian period, whereby a longer period reduces the extent of dawn anticipation by morning‐phased reporters, and a shorter period leads to more obvious anticipation of dawn (Dodd *et al*., 2014). However, the circadian period of *SIG5::LUCIFERASE* was not longer under B light than under R + FR + B conditions (Fig. 4d,h), suggesting that period differences might not explain this variation in dawn anticipation. Another possibility is that increased photosynthetic energy availability in R + FR + B light relative to other treatments caused the *SIG5* promoter to assume an earlier phase, because increased energy availability can shorten the circadian period (Haydon *et al*., 2013). We speculate that the anticipation of dawn by *SIG5* might be important to ensure appropriate rates of PSII D2 protein accumulation before the onset of photosynthesis. However, to better understand the adaptive significance of these results, it will be important to determine the contribution of the circadian oscillator to the dynamics of this pathway under lighting conditions more representative of natural environments.

Differences between the transcriptional response of *SIG5* to specific light conditions during acute induction and circadian free‐run provide information about the contribution of circadian regulation to the functioning of this pathway under light–dark cycles, and about the role of specific light conditions around dawn. *SIG5* responded strongly to R + FR light in dark‐adapted plants (Fig. 1a,b) and under light–dark cycles (Fig. 5a), suggesting that, in nature, R + FR light might be an important regulator of *SIG5* around dawn. In comparison, B light and cry1 help to maintain *SIG5* transcript accumulation longer term, such as during the circadian free‐run (Fig. 4c,d,h) and the second half of the photoperiod (Fig. 5). Therefore, circadian regulation contributes to *SIG5* promoter activity during light–dark cycles. Under light–dark cycles, circadian regulation might be particularly important for gating the responses of *SIG5* to transient changes in light conditions in order to maintain optimum synthesis of PSII D2 (Noordally *et al*., 2013).

Regulators of *SIG5* transcription in response to light include ELONGATED HYPOCOTYL5 (HY5) and HY5 HOMOLOG (HYH), which act redundantly to regulate SIG5 transcript accumulation (Nagashima *et al*., 2004; Brown & Jenkins, 2008; Mellenthin *et al*., 2014). Abscisic acid also upregulates *SIG5* transcripts, but may be without effect on chloroplast *psbD* under the same conditions (Yamburenko *et al*., 2015). Although there are a variety of other light‐ and circadian‐regulated *cis* elements within the *SIG5* promoter (Noordally *et al*., 2013; Mellenthin *et al*., 2014), it is less clear which pathways underlie the circadian regulation of *SIG5*. For example, the high mean level of SIG5 promoter activity in B + R + FR compared with B light (Fig. 4a) might reflect convergence on the SIG5 promoter of distinct signals that regulate its activity. This could mean that, under certain lighting conditions, basal *SIG5* promoter activity might be increased to a point at which its circadian amplitude becomes reduced or masked. Although circadian oscillations of the *SIG5* promoter and *SIG5* transcript abundance are morning phased, the dawn‐phased oscillator component CIRCADIAN CLOCK ASSOCIATED1 (CCA1) does not appear to bind the *SIG5* promoter (Nagel *et al*., 2015).

### The response of SIG5 to the proportions of red and far red light may involve photosynthetic retrograde signals

The transcriptional response of *SIG5* and *psbD* BLRP to the relative proportions of R and FR light was atypical of regulation by phytochrome alone (Fig. 3). As FR > *c*. 700 nm has insufficient quantum energy to drive oxygenic photosynthesis (Chen & Blankenship, 2011), we reasoned that photosynthetic signals might contribute to this R : FR response because SIG5 transcription can be regulated by photosynthesis (Mellenthin *et al*., 2014). Moreover, there was little alteration in *SIG5* promoter activity or transcript abundance across much of the R : FR range that induces shade avoidance responses, except for R : FR conditions typical of deeper shade (R : FR below *c*. 0.2) (Smith, 1982). *sig5*‐3 has been reported to have shorter hypocotyls and smaller cotyledons than the wild‐type in either constant R or FR light, 4 d after germination (Khanna *et al*., 2006). This was interpreted as a cell expansion defect rather than a photomorphogenic phenotype, potentially caused by increased sensitivity of *sig5*‐3 to light‐induced damage (Khanna *et al*., 2006), which is consistent with the slow recovery of PSII photochemistry after exposure of *sig5* mutants to high light (Nagashima *et al*., 2004).

One interpretation of the response of *SIG5* to R and FR light is that, when a large proportion of light is FR, little energy is available to drive oxygenic photosynthesis. Under these conditions, the photosynthetic signal that regulates SIG5 transcription is weak, inhibiting the phyA signal and suppressing *SIG5* transcription (Figs 2c, 4b). This would also explain the insensitivity of *SIG5* to DCMU under conditions of predominantly FR light (Fig. 3b), because DCMU inhibits photosynthetic electron transport from PSII to PQ, which decreases substantially under predominantly FR light in which PSII is less activated than PSI.

Interestingly, the differing R : FR response profiles of *SIG5* and *psbD* BLRP suggest that there is post‐translational regulation of SIG5 activity (Fig. 3). This is also supported by our findings that *SIG5* transcripts were not induced by R + FR light in the *phyA* mutant, whereas *psbD* BLRP was induced by higher intensity R + FR light in *phyA* (Fig. 2d); phyA‐mediated activation of *psbD* BLRP by R + FR light required phyB, whereas phyA‐mediated activation of *SIG5* did not require phyB (Fig. S4b); and B light induced *SIG5* through either cry1 or cry2, whereas B light induction of *psbD* BLRP required both cry1 and cry2 (Fig. S4c). Post‐translational regulation might involve phosphorylation of SIG5 protein on one or more of its predicted serine/threonine phosphorylation sites, similar to redox‐dependent regulation of SIG1 and chloroplast transcription by PLASTID TRANSCRIPTION KINASE (PTK) and CHLOROPLAST SENSOR KINASE (CSK) (Baena‐González *et al*., 2001; Shimizu *et al*., 2010). Another possibility is that there is light and/or redox regulation of SIG5 chloroplast protein import (Küchler *et al*., 2002; Hörmann *et al*., 2004). In this context, future analysis of SIG5 protein biology may be informative. Although there could also be SIG5‐independent regulation of *psbD* BLRP, this is not supported by an analysis of *psbD* transcripts accumulating from all TSSs of the chloroplast *psbDC* operon (Nagashima *et al*., 2004). In future, it will be informative to determine whether the regulation of chloroplast transcription by sigma factors contributes to photosynthetic adaptation to shade under light conditions more representative of natural environments, and to investigate the nature of the photosynthetic retrograde signal that regulates SIG5 in response to changing light conditions.

## Conclusions

The regulation of photosynthesis gene expression by sigma factors in response to light appears to be conserved throughout photosynthetic life. It is possible that this signalling pathway evolved as an adaptation to light stress. In cyanobacteria, sigma factors have an important role in maintaining optimum growth under high light conditions by regulating the expression of photosystem components (Hanaoka & Tanaka, 2008; Pollari *et al*., 2009). This function appears to have been conserved following the endosymbiosis that led to the evolution of chloroplasts, because the regulation of chloroplast genes by sigma factors is important to maintain photosynthetic efficiency under very high light in Arabidopsis (Nagashima *et al*., 2004). Our data suggest that, during evolution, this light stress response pathway has become rewired to also underpin subtle and sophisticated responses to the light environment by the integration of a conserved signalling pathway with higher plant photoreceptor systems, retrograde signalling and the circadian clock.

## Author contributions

F.E.B., Z.B.N., K.A.F. and A.N.D. conceived and designed the experiments. F.E.B. and Z.B.N. performed the experiments. S.J.W. prepared some transgenics. K.A.A. provided essential technical assistance (Fig. S3). F.E.B., Z.B.N., K.A.F. and A.N.D. interpreted the data and wrote the paper.

## Supporting information

Please note: Wiley Blackwell are not responsible for the content or functionality of any Supporting Information supplied by the authors. Any queries (other than missing material) should be directed to the *New Phytologist* Central Office.

**Fig. S1** Initial circadian period characterization of multiple luciferase lines.
**Fig. S2** Spectra of light treatments used in this study.
**Fig. S3** Efficacy of 3‐(3,4‐dichlorophenyl)‐1,1‐dimethylurea (DCMU) and norflurazon under our experimental conditions.
**Fig. S4** Analysis of photoreceptors involved in SIGMA FACTOR5 (SIG5)‐mediated regulation of chloroplast *psbD* blue light‐responsive promoter (BLRP).
**Fig. S5** Circadian regulation of *SIG5::LUCIFERASE* by light quality and photoreceptors.
**Fig. S6** Circadian regulation of *SIG5::LUCIFERASE* in the Col‐0 background by light quality.
