# Supplementary material for: Integration of light and circadian signals that regulate chloroplast transcription by a nuclear‐encoded sigma factor
Source: New Phytol. 2016 Sep 15;213(2):727–38. doi: 10.1111/nph.14176 (PMC5215360; doi:10.1111/nph.14176)
Supplement: Supplementary file 1 — Fig. S1 Initial circadian period characterization of multiple luciferase lines. Fig. S2 Spectra of light treatments used in this study. Fig. S3 Efficacy of 3‐(3,4‐dichlorophenyl)‐1,1‐dimethylurea (DCMU) and norflurazon under our experimental conditions. Fig. S4 Analysis of photoreceptors involved in SIGMA FACTOR5 (SIG5)‐mediated regulation of chloroplast psbD blue light‐responsive promoter (BLRP). Fig. S5 Circadian regulation of SIG5::LUCIFERASE by light quality and photoreceptors. Fig. S6 Circadian regulation of SIG5::LUCIFERASE in the Col‐0 background by light quality. [file NPH-213-727-s001.pdf]

Belbin *et al*. Integration of light and circadian signals that regulate chloroplast transcription by a nuclear-encoded sigma factor

Supporting Material Figures S1 – S6.

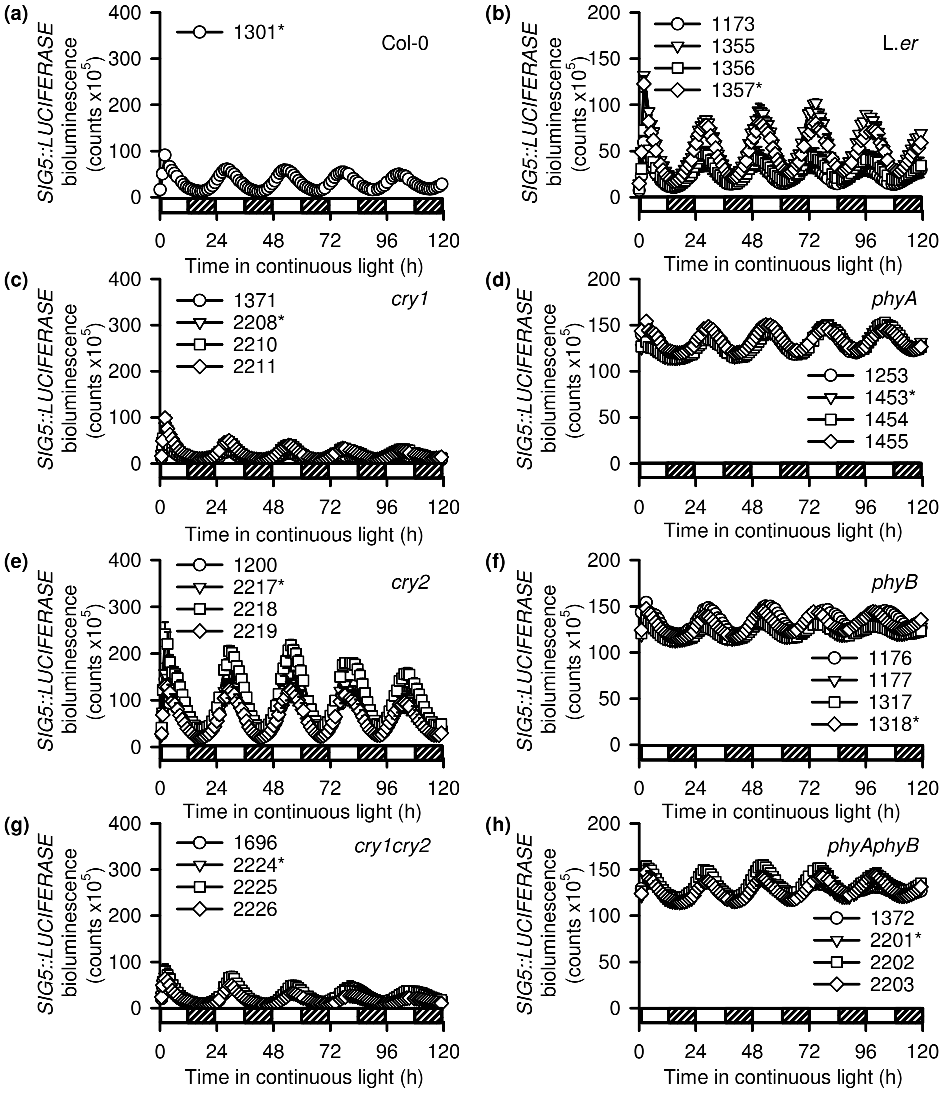

(i)

Genotype / Line	Mean period (h, ± s.e.m)	Genotype / Line	Mean period (h, ± s.e.m)
Col-0 *[a]	24.3 ± 0.2	L. *er.* 1356	23.4 ± 0.2
cry1 2210	24.6 ± 0.1	L. *er.* 1357 *	23.4 ± 0.1
cry1 2211	25.6 ± 0.3	L. *er.* 1173	23.0 ± 0.1
cry1 2208 *	24.3 ± 0.2	L. *er.* 1355	23.1 ± 0.1
cry1 1371	24.8 ± 0.2	*phyA* 1455	25.1 ± 0.1
cry2 1206	24.8 ± 0.1	*phyA* 1454	24.9 ± 0.2
cry2 2217 *	24.7 ± 0.3	*phyA* 1453 *	24.9 ± 0.3
cry2 2218	24.6 ± 0.1	*phyA* 1253	24.9 ± 0.1
cry2 2219	24.4 ± 0.1	*phyB* 1176	23.7 ± 0.1
cry1 cry2 2226	25.8 ± 0.3	*phyB* 1177	24.3 ± 0.1
cry1 cry2 2225	25.7 ± 0.2	*phyB* 1317	23.8 ± 0.1
cry1 cry2 2224 *	25.3 ± 0.1	*phyB* 1318 *	23.3 ± 0.2
cry1 cry2 1696	25.8 ± 0.3	*phyA phyB* 2201 *	23.5 ± 0.1
		phyA phyB 1372	23.8 ± 0.4
		phyA phyB 2202	24.0 ± 0.2
		phyA phyB 2203	23.8 ± 0.2

Figure S1. Initial characterization of luciferase reporter lines for subsequent experimentation. (a-h) *SIG5::LUCIFERASE* bioluminescence timecourses from 4 transgenic lines of each mutant/background accession; (i) circadian period of *SIG5::LUCIFERASE* in each line. A

representative line of each accession (indicated by asterisk) was selected for subsequent work. [a]Col-0 *SIG5::LUCIFERASE* was published previously (Noordally *et al*. 2013), so was not characterized here. Characterization occurred under a combination of red and blue light (25 µmol m^{-2} s^{-1} of each), with timecourses starting 11 days after seedling germination. Data are mean ± s.e.m; n = 3.

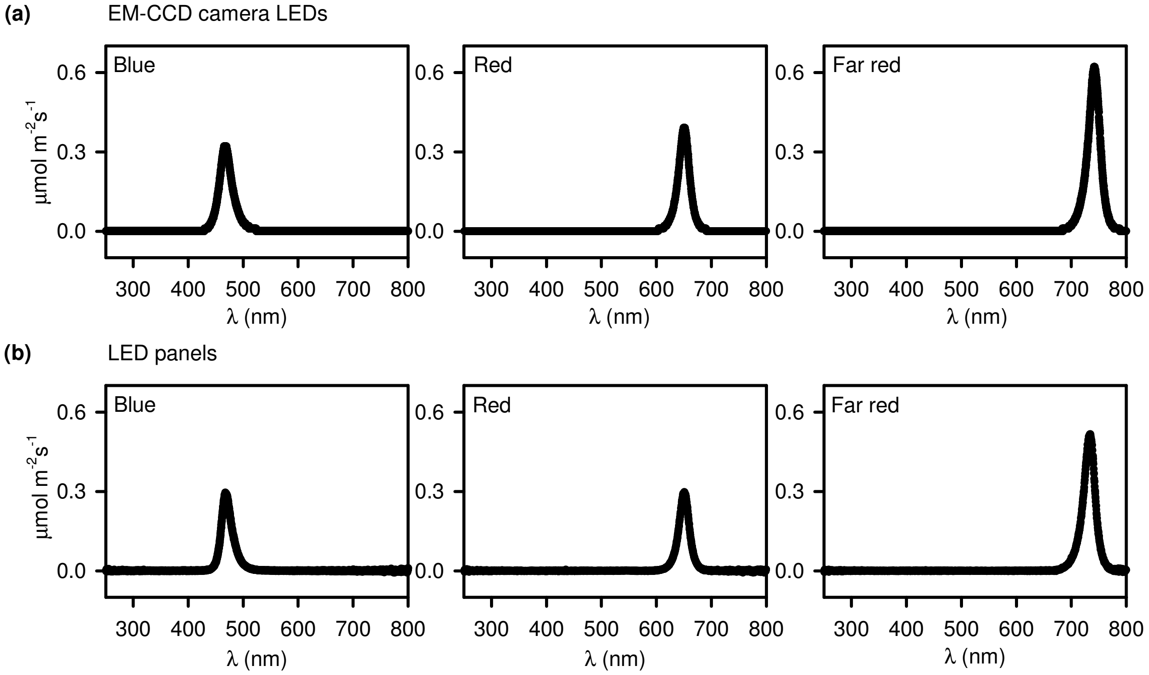

Figure S2. Light treatment spectra. Spectra of blue, red and far red LEDs of (a) Photek LB-1 LED panels in EM-CCD camera (used in Fig. 1a (luciferase imaging experiment), Fig. 1b and c, Fig. 2, Fig. 3, Fig. 4, Fig. 5, Fig S1, Fig. S3a and Fig. S4, Fig. S5, Fig. S6); (b) LED panels in plant growth chambers (Fig. 1a (*SIG5* and *psbD* BLRP transcript experiments) and Fig. S4).

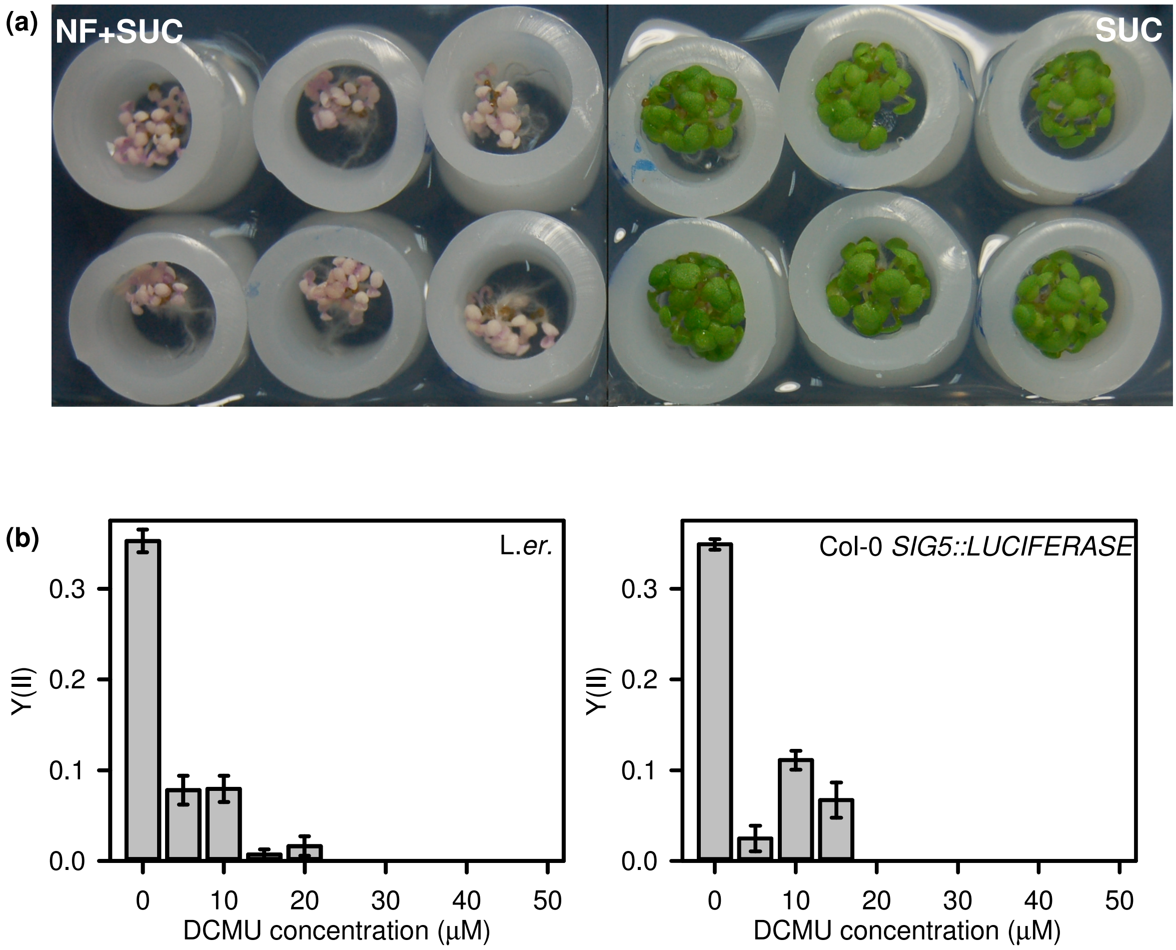

Figure S3. Efficacy of photosynthetic inhibitors under our experimental conditions. (a) 5μM norflurazon (NF) produced photobleached living Arabidopsis seedlings when added to media containing 1% sucrose. Seedlings were constrained within the same type of plastic ring used for bioluminescence imaging. (b) Determination of minimum effective dose of DCMU (20μM) that inhibited photosynthesis, measured by chlorophyll fluorescence, in L. *er.* wild type and also Col-0 *SIG5::LUCIFERASE*, where DCMU was combined with 5 mM luciferin. Y(II) provides a measure of the effective quantum yield of PSII. (a) Photography and (b) chlorophyll fluorescence analysis was conducted 11 days after germination, in seedlings cultivated as described in the Materials and Methods section for bioluminescence imaging. Data are mean ± s.e.m; n = 6.

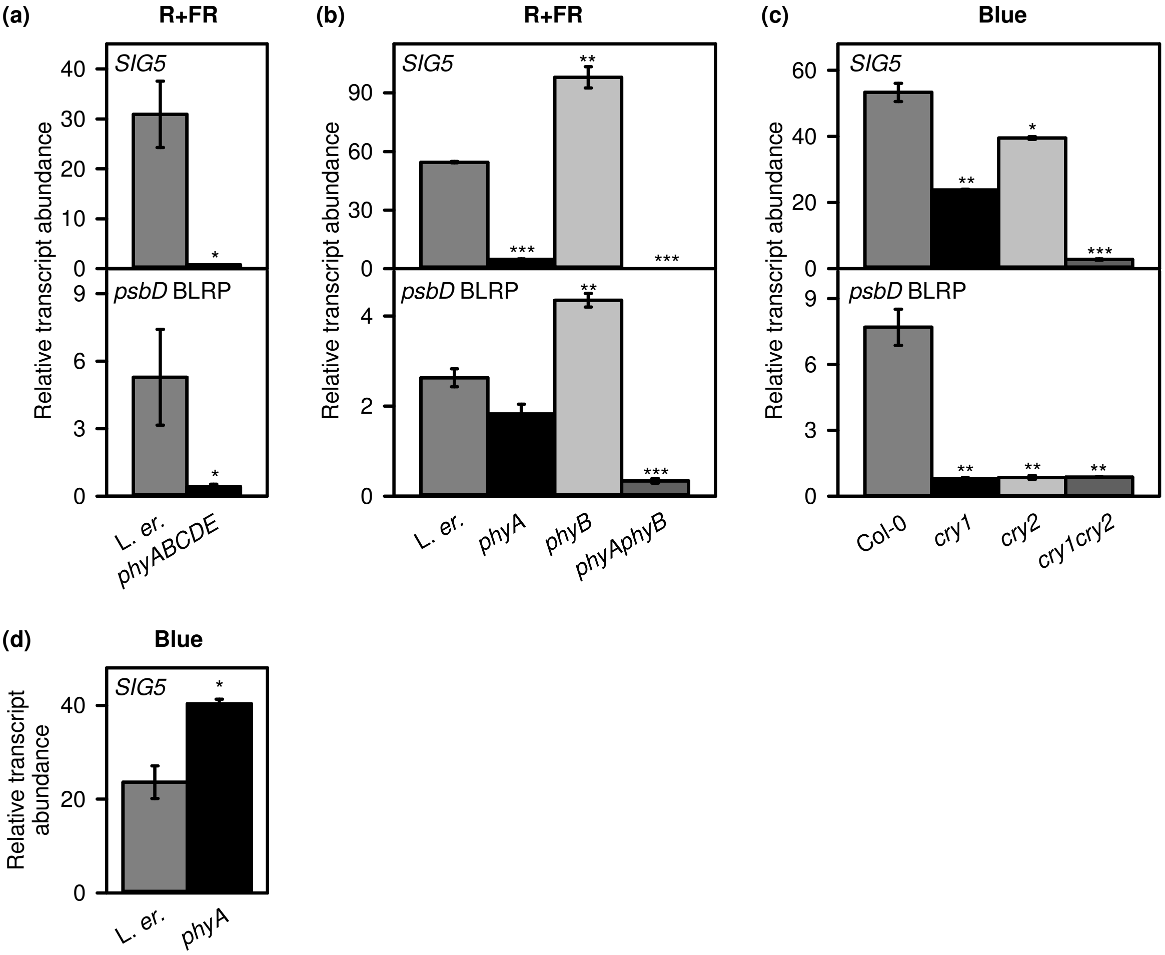

Figure S4. Investigation of photoreceptors involved in SIG5-mediated regulation of chloroplast *psbD* BLRP. (a, b) Phytochromes are required for *SIG5* and *psbD* BLRP induction by R+FR (R:FR 0.7). (c) cry1 and cry2 are necessary for transient *SIG5* and *psbD* BLRP induction by B. (d) Induction of *SIG5* transcripts by B is altered in *phyA*. Significance derived from t-test comparing WT and mutants. *** = $p < 0.001$; ** = $p < 0.01$; * = $p < 0.05$. Data are mean ± s.e.m; n = 2 or 3.

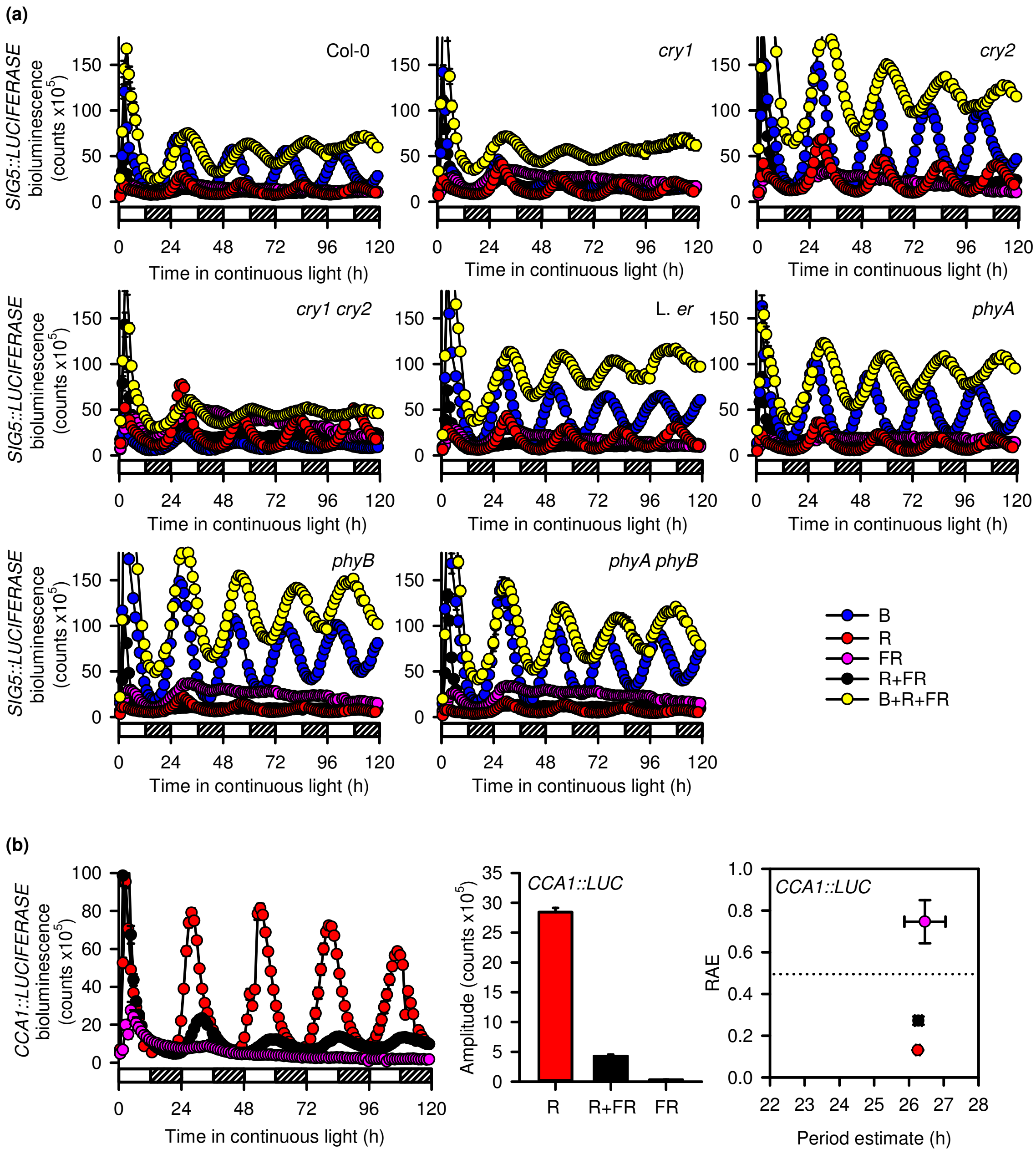

Figure S5. Circadian regulation of *SIG5::LUCIFERASE* by light quality and photoreceptors. (a) *SIG5::LUCIFERASE* bioluminescence timecourses under five light regimes and six photoreceptor mutant genotypes that were analyzed to produce Fig. Xd-h. (b) Circadian dynamics of *CCA1::LUCIFERASE* under continuous red, far red, or red plus far red light. Hatched bars on *x* axes indicate subjective darkness. Data are mean ± s.e.m; n = 4; B=blue light, R=red light, FR=far red light, R:FR in R+FR was 0.7.

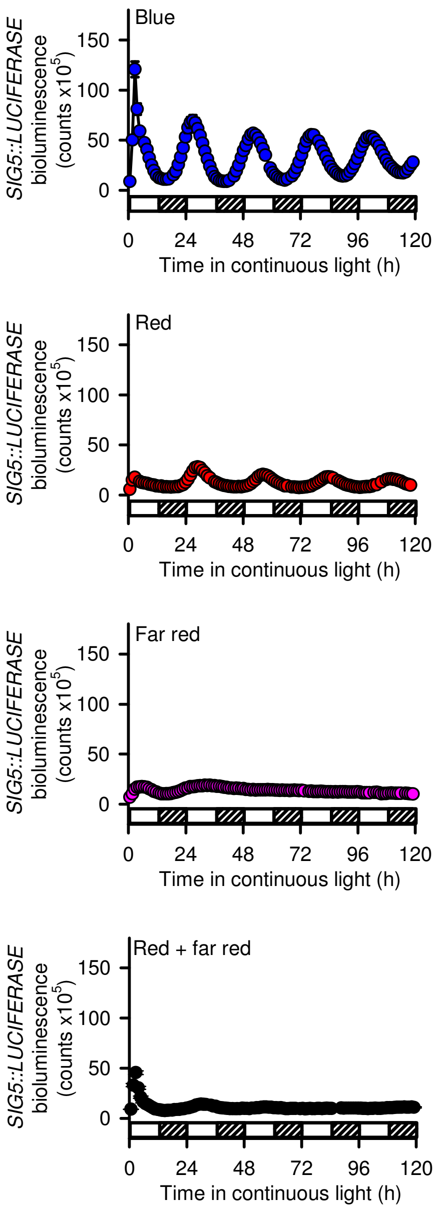

Figure S6. Circadian regulation of *SIG5::LUCIFERASE* by light quality. *SIG5::LUCIFERASE* bioluminescence timecourses under four light regimes. These are data from Fig. 4a plotted on independent panels for clarity. Data are mean ± s.e.m.; n = 4.

Reference

Noordally ZB, Ishii K, Atkins KA, Wetherill SJ, Kusakina J, Walton EJ, Kato M, Azuma M, Tanaka K, Hanaoka M, et al. 2013. Circadian control of chloroplast transcription by a nuclear-encoded timing signal. *Science* 339: 1316-1319.
